# Among the mimickers of Stevens-Johnson syndrome: A case of anasarca-induced skin desquamation

**DOI:** 10.1016/j.jdcr.2024.06.029

**Published:** 2024-07-06

**Authors:** Rachel Manci, William Guo, Matthew Chen, Jeremy Hugh, Katherine Siamas

**Affiliations:** Department of Dermatology, Stony Brook University Medical Center, Stony Brook, New York

**Keywords:** anasarca, case report, skin desquamation, Stevens-Johnson syndrome, toxic epidermal necrolysis

## Introduction

Anasarca-induced desquamation is a relatively benign condition resulting in cutaneous skin sloughing of large surface areas due to underlying subcutaneous edema. The desquamation is localized to the stratum corneum, and there is no eventual sequelae of epidermal necrosis. Although the pathophysiology of anasarca-induced desquamation is not well elucidated, it is hypothesized that this condition is a diffuse form of edema blisters, which develop when the capillary filtration rate exceeds lymphatic drainage for a sufficient period of time.^1^ Cardiac, hepatic, or renal disease are common causes of anasarca, and the subsequent desquamation is likely a product of the rapid fluid shifts and sudden weight gain that can be caused by these conditions.^1^^,^^2^

Although anasarca-induced desquamation is an uncommon occurrence, its resemblance to other more critical illnesses such as Stevens-Johnson syndrome (SJS) or toxic epidermal necrolysis (TEN) can pose a diagnostic challenge. Here we present a case of anasarca-induced desquamation that initially mimicked the clinical presentation of SJS/TEN, thus prompting an initial rapid diagnostic workup.

## Case report

A 60-year-old woman with a past medical history of hypertension, hyperlipidemia, and sarcoidosis presented to the hospital after having a fall at home and subsequently being immobilized on the ground for 2 days before being found. Upon arrival, the patient was tachycardic and hypotensive. Laboratory results revealed a leukocytosis (39.44 K/μL; normal 4-10 L/μL), an elevated lactate level (3.4 mmol/L; normal 0.5-2.0 mmol/L), altered kidney function (creatinine 2.65 mg/dL; normal 0.5-1.20 mg/dL, blood urea nitrogen 55 mmol/L; normal 21-31 mmol/L), and an elevated creatine kinase level (527 IU/L; normal 26-174 IU/L). Given that the patient was demonstrating features of systemic inflammatory response syndrome, she was pan-cultured and started on broad-spectrum antibiotics (vancomycin and ceftriaxone). Blood and urine cultures returned negative. She received copious amounts of intravenous (IV) fluids due to concern for rhabdomyolysis and acute kidney injury, which resulted in generalized edema. Throughout the initial few days of her hospitalization, her antimicrobial regimen was changed several times, and she ultimately became exposed to anidulafungin, meropenem, vancomycin (IV and perioral formulations), cefepime, and ceftriaxone.

**Fig 1:**
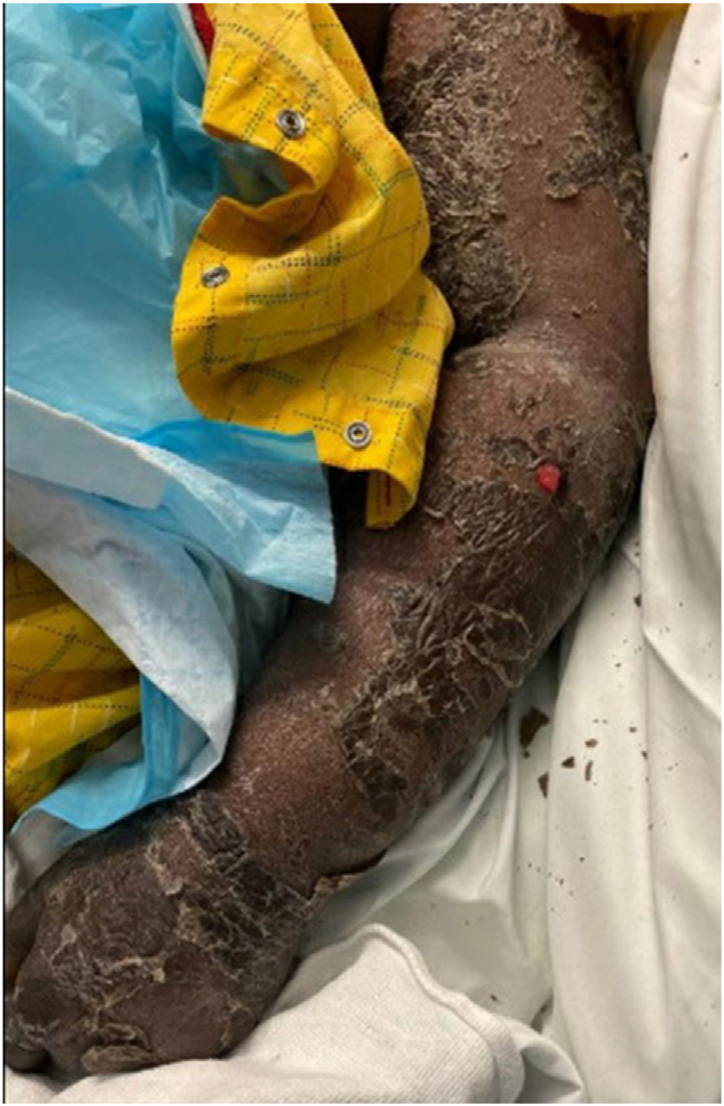
Superficial desquamation also with a focal erosion on the left forearm.

**Fig 2:**
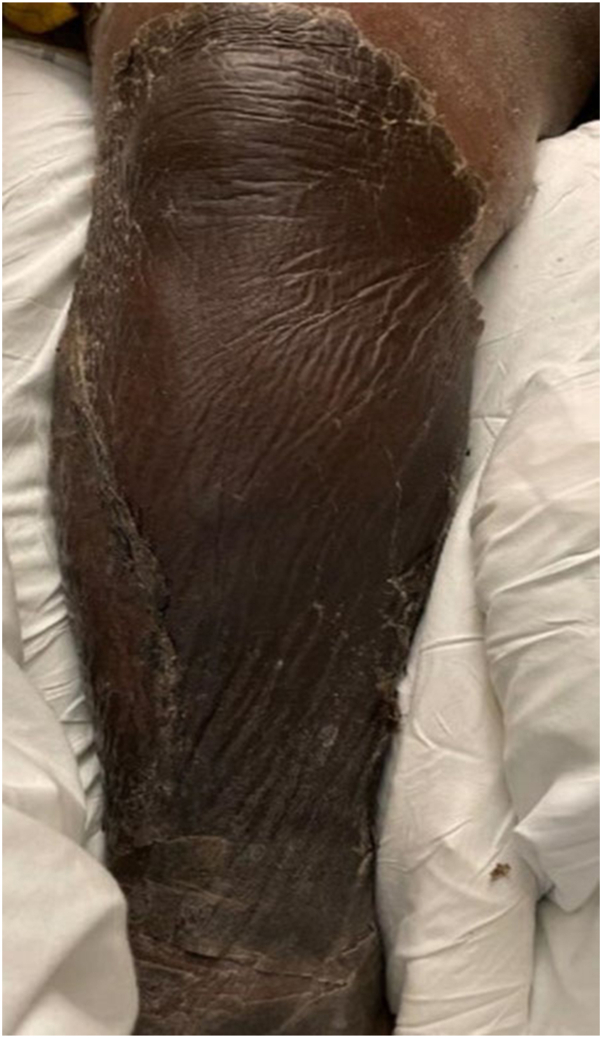
Superficial desquamation of the lower portion of the right leg.

**Fig 3:**
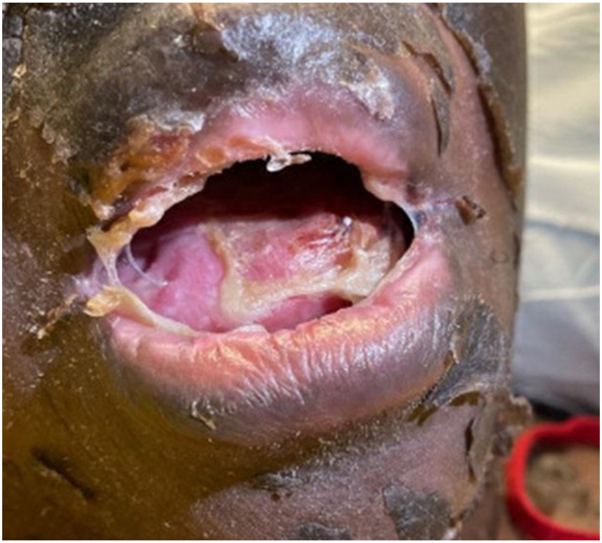
Oral mucosal sloughing with accompanying desquamation of the facial skin.

Seven days after the initial presentation, the patient experienced superficial skin desquamation that began on the lower extremities, and progressed over the course of a few days to also involve the anterior/posterior aspect of the trunk, upper extremities, and the head/neck region. The patient had superficial skin sloughing and desquamation of her entire body with one focal area of superficial erosion (Figs 1 and 2). The patient also had sloughing of the facial skin and oral mucosa (Fig 3). She also had ocular involvement with bilateral conjunctival injection. Nikolsky sign was negative. The patient stated she had both skin and mucosal pain at this time.

**Fig 4:**
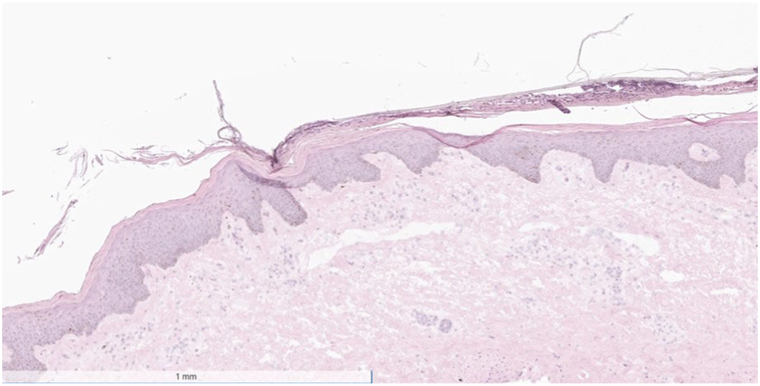
Skin histology slide showing plate-like scale-crust overlying normal epidermis with a chronic inflammatory cell infiltrate in the papillary dermis.

Concern for TEN was high, and 2 broad shave biopsies were performed emergently—1 for fresh frozen section and 1 that was split for permanent hematoxylin-eosin and direct immunofluorescence microscopy. The differential diagnosis at this time included TEN, anasarca-induced desquamation, immunobullous disorders such as pemphigus vulgaris, Kwashiorkor, and staph scalded skin syndrome. The biopsies flash frozen and hematoxylin-eosin were taken of lesional skin in an area of erosion, whereas the direct immunofluorescence was taken from perilesional skin. Frozen sections revealed plate-like scale-crust overlying normal epidermis with a chronic inflammatory cell infiltrate in the papillary dermis. Permanent sections again failed to reveal SJS/TEN specific features, but did reveal parakeratosis with a few inflammatory cells in the stratum corneum (Fig 4). The patient’s family endorsed that she ate a balanced diet at home and that her albumin level was normal at 4.2 g/dL (normal 3.5-4.8 g/dL). Direct immunofluorescence microscopy was negative for IgA, IgG, and C3. In this clinical setting, the patient was ultimately diagnosed with generalized edema bullae/anasarca-induced desquamation.

## Discussion

Anasarca-induced desquamation is a more generalized form of edema bullae.^1^^,^^2^ Ultimately, our patient’s fall and the resulting prolonged period of time that she spent immobilized led to rhabdomyolysis further complicated by an acute kidney injury and temporary acute renal failure. Early and aggressive fluid replacement is the mainstay of treatment to prevent and treat kidney irregularities caused by rhabdomyolysis,^3^ which is exactly how our patient had been treated. Fortunately, her kidney function recovered and she did not necessitate dialysis, but nonetheless, she had received copious amounts of IV fluids during the aggressive fluid resuscitation. This build-up of fluid resulted in diffuse anasarca, and the rapid fluid shifts ultimately resulted in the desquamation of almost her entire body. Thankfully, our patient recovered and had no long-term cutaneous side effects, although the initial presentation was quite alarming in appearance.

To our knowledge, this phenomenon has only been reported one other time in the literature.^1^ Kou et al^1^ also reported total body desquamation in a patient who received IV fluid resuscitation in the setting of acute renal failure. Contrasting to Kou et al’s^1^ patient, our patient had superficial desquamation of her conjunctiva and oral mucosa. Consults from ophthalmology and ear-nose-throat confirmed there was no underlying erosion or ulceration, as well as no lasting damage to the mucosae. This patient was noted to have a 40-pound weight gain over the course of 5 days.^1^

For both edema blisters and anasarca-induced desquamation, the histopathologic level of split within the epidermis is not well described in the literature. Kou et al^1^ performed a punch biopsy on their anasarca-induced desquamation patient and revealed an intracorneal split with desquamation of the stratum corneum. Our patient also had similar findings. This is a significant difference when compared with SJS and TEN, as there is more vast keratinolysis and necrosis diffusely within the epidermis.^4^ This correlates with the resultant clinical features observed in SJS/TEN and anasarca-induced desquamation, with the prior having underlying erosions and ulceration beneath the skin sloughing and the latter having normal keratinized skin beneath the shedding layer.

## Conclusion

Although alarming, diffuse desquamation may represent a relatively benign skin condition, as seen in our patient. Other more serious mimickers such as SJS and TEN must be ruled out, but understanding the entirety of the differential diagnosis is imperative for effective clinical decision making. Although rare, one must consider anasarca-induced skin desquamation in the appropriate clinical context.

## Conflicts of interest

None disclosed.
